# Congenital Insensitivity to Pain with Anhidrosis: A Case Report

**DOI:** 10.7759/cureus.48294

**Published:** 2023-11-05

**Authors:** Noura M Sulaiman, Eram Alyahya

**Affiliations:** 1 Pediatric Dentistry, Prince Sultan Military Medical City, Riyadh, SAU

**Keywords:** comprehensive care, dental manifestations, interdisciplinary approach, clinical complexities, genetic disorder, congenital insensitivity to pain with anhidrosis (cipa)

## Abstract

Hereditary sensory and autonomic neuropathy type 4 (HSAN4), or congenital insensitivity to pain with anhidrosis (CIPA), is a rare autosomal recessive disorder caused by mutations in the NTRK1 gene, resulting in pain insensitivity, anhidrosis, and temperature dysregulation. This report focuses on oral manifestations in an 11-year-old girl with CIPA, highlighting the need for early intervention and comprehensive care. The patient had a history of recurrent oral injuries and an unexplained fever, with a confirmed HSAN4 diagnosis through genetic analysis. Clinical features included pain insensitivity, anhidrosis, and intellectual disability. Dental history revealed emergency care, suboptimal oral hygiene, early tooth loss, and infections. Extra-oral examination showed nail-biting and injuries, while intra-oral assessment revealed ulcers and scars. Radiographic evaluation indicated mandibular alveolar bone thinning and periapical lesions in the lower incisors. This case emphasizes the complex challenges of CIPA, including pain insensitivity, recurring fever episodes, and self-inflicted injuries. Early diagnosis and specific dental care are vital to prevent orofacial trauma, necessitating a proactive interdisciplinary approach for comprehensive care.

## Introduction

Hereditary sensory and autonomic neuropathy type 4 (HSAN4), also known as congenital insensitivity to pain with anhidrosis (CIPA), is an exceedingly rare genetic disorder belonging to the category of hereditary sensory and autonomic neuropathies (HSANs) [1]. It is characterized by the inability to perceive pain, impaired temperature regulation, and anhidrosis. The underlying genetic defect associated with CIPA involves mutations in the neurotrophic tyrosine kinase receptor 1 (NTRK1) gene on chromosome 1, a receptor for nerve growth factor, thus leading to aberrant neural development and signaling pathways [2]. For this reason, patients with CIPA will demonstrate an absence of unmyelinated fibers and a loss of small, myelinated fibers [3,4].

CIPA is an exceedingly rare disorder, with a reported incidence of one in 125 million [5]. The disease shows geographic variability, which may be attributed to founder mutations and consanguineous marriages [6]. The condition exhibits an autosomal recessive inheritance pattern, necessitating the presence of two mutated alleles for phenotypic expression [7]. The majority of affected individuals originate from consanguineous unions, emphasizing the importance of genetic counseling in at-risk populations.

CIPA is primarily caused by mutations in the NTRK1 gene, localized to chromosomes 1q21-q22. NTRK1 encodes the high-affinity nerve growth factor receptor, TrkA, which is crucial for the survival and differentiation of sensory and autonomic neurons [8]. Mutations in NTRK1 result in defective neural crest cell migration, impaired neurite outgrowth, and altered axon guidance. Disruption of neural development pathways leads to the absence of nociceptive and thermoregulatory neurons, culminating in the hallmark features of CIPA [9].

Clinically, CIPA is characterized by a triad of symptoms: insensitivity to pain, anhidrosis, and temperature dysregulation [10]. Notably, tactile sensation, lacrimation, and salivation are not affected [3,4]. Affected individuals are prone to accidental and self-inflicted injuries, including intra-oral injuries, and infections due to their inability to perceive pain. Anhidrosis contributes to the inability to sweat, leading to increased susceptibility to hyperthermia. The diagnosis is based on clinical evaluation, genetic testing, and a nerve biopsy. Genetic analysis of the NTRK1 gene is essential for confirming the diagnosis and identifying the specific mutation [11].

The management of CIPA primarily focuses on alleviating symptoms and preventing complications. Since there is no curative treatment available, interventions aim to enhance the quality of life for affected individuals [12]. Strategies include vigilant wound care, pain avoidance education, and proactive measures to prevent hyperthermia. Ongoing research is exploring potential therapeutic avenues, including gene therapy and pharmacological interventions targeting neural development pathways.

This case presentation discusses the clinical characteristics of an 11-year-old female patient diagnosed with CIPA. The patient's complex medical history and dental issues were thoroughly assessed to understand the implications of CIPA on her overall health and oral well-being. The purpose of this report is to expand the current knowledge and understanding of the oral manifestations encountered in patients with CIPA and the necessity of early intervention and conservative treatment.

## Case presentation

The subject, an 11-year-old girl, was referred to the pediatric dental clinic of Prince Sultan Military Medical City in Riyadh, Saudi Arabia, due to a history of recurrent traumatic injuries to the lower lip and oral cavity. Her parents have reported multiple instances of unexplained fever since infancy. A comprehensive genetic work-up at eight months of age revealed a homozygous variant in the NTRK1 gene, leading to a confirmed diagnosis of HSAN4. The patient's clinical presentation included insensitivity to pain, anhidrosis, and intellectual disability. She experienced recurrent episodes of fever and wounds that led to cellulitis, necessitating hospitalization. The patient's intellectual quotient (IQ) was assessed, yielding a score of 61, and she received education in a special school.

Dental history

The patient had no established dental care at home and only sought emergency dental care due to pain and swelling. Her oral hygiene practices were suboptimal, with irregular toothbrushing (four times a week) and no use of dental floss. Her dental history revealed a recurrent pattern of dental infections leading to extra-oral facial swelling over the past four years in the form of cellulitis. The cellulitis was caused by dental infections. The infected teeth were carious. Unfortunately, due to the patient's lack of cooperation, no dental treatment was done in the clinic other than the antibiotic prescription. The patient had undergone two dental rehabilitation procedures under general anesthesia, the most recent one occurring a year prior to presentation. These interventions encompassed multiple restorations and extractions of both primary and permanent teeth.

Clinical characteristics

**Figure 1:**
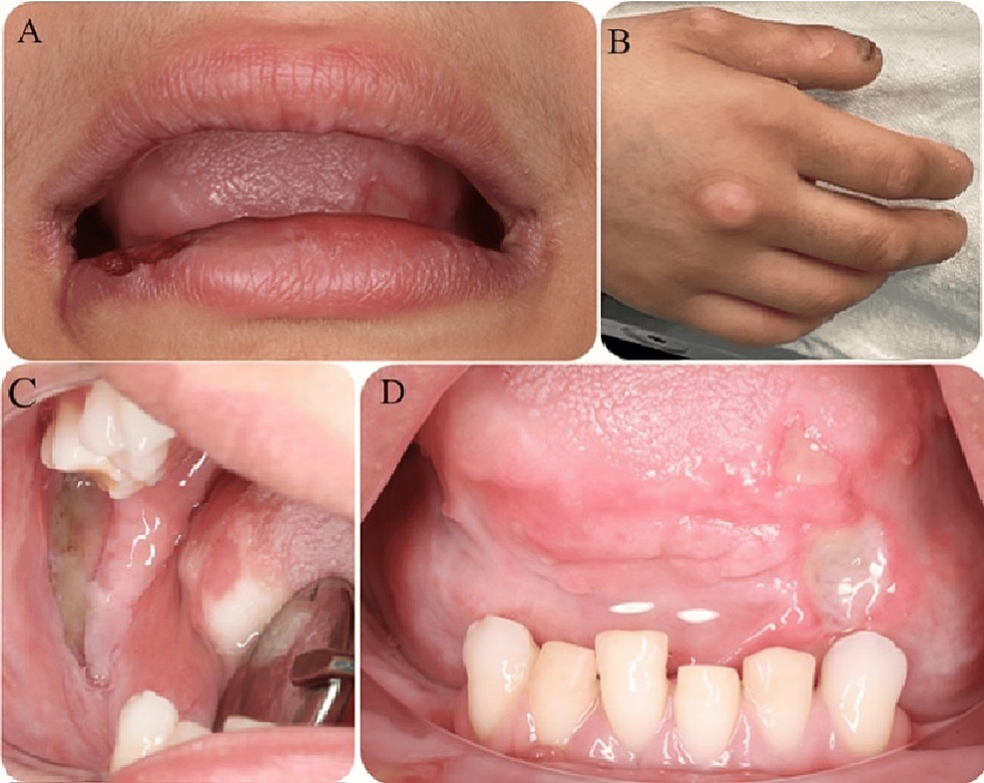
Multiple photographs show the multiple injuries (A) ulceration and crusting in the lower lip; (B) biting marks on the fingers; (C,D) extensive ulcers in the retromolar pad and tongue, respectively

The patient's general demeanor was noted to be cheerful and friendly during the initial dental examination. Developmental delay and dry skin were evident in her overall appearance. Extra-oral examination revealed self-inflicted nail-biting behavior, leading to nearly absent fingernails. Biting marks on fingers and multiple injuries on the toes and soles of her feet were observed (Figure 1B). Intra-oral examination unveiled ulceration and crusting on the lower lip's left side (Figure 1A), as well as multiple traumatic ulcers on the tongue's ventral and dorsal aspects. Extensive ulcers were identified on the right buccal mucosa and retromolar pad region (Figures 1C, 1D). Additionally, old scars and bite marks were evident on the buccal mucosa. Limited mouth opening was attributed to the presence of thick fibrous scar tissue on her cheek due to repeated biting.

**Figure 2:**
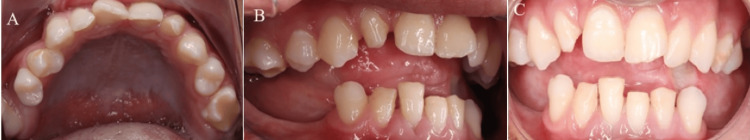
Intra-oral photographs A) Upper occlusal view; B) lateral view; C) frontal view; D) lateral view; E) lower occlusal view

**Figure 3:**
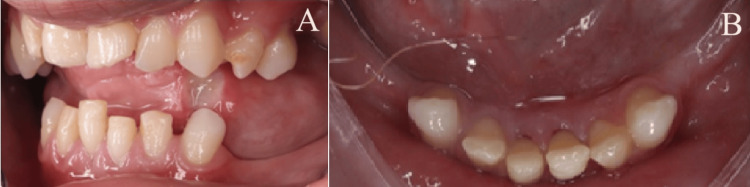
Intra-oral photographs A) lateral view; B) lower occlusal view

Furthermore, the patient is currently in the permanent dentition stage, with multiple missing teeth (Figure 2 and 3). These missing teeth result from a combination of congenitally missing teeth and extractions of unrestorable teeth. These extractions were necessitated by factors such as caries and Grade III mobility. Notably, these dental procedures were performed during two dental rehabilitation sessions conducted under general anesthesia. Importantly, the maxillary teeth present sound conditions. The permanent mandibular incisors exhibit no signs of caries; however, Grade II mobility was detected in these incisors, implying a certain degree of tooth movement. Unfortunately, a cold test to assess dental pulp vitality was unfeasible due to the patient's medical condition. Despite this limitation, the mother consistently denied any history of trauma that she was aware of, suggesting that the tooth mobility may be connected to the underlying neuropathic characteristics of CIPA.

Radiographic evaluation

**Figure 4:**
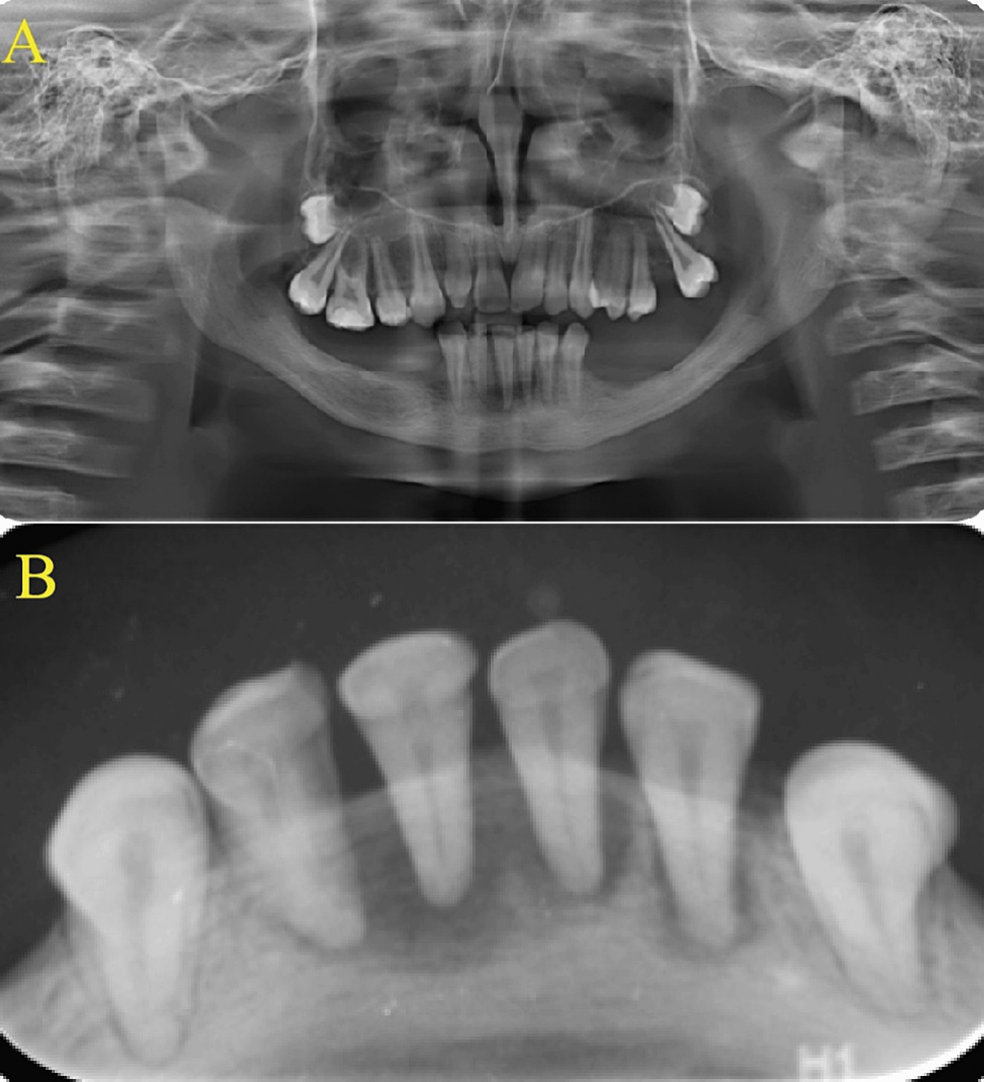
Radiographic assessments (A) panoramic radiograph; (B) periapical radiographs of lower anterior teeth

Radiographic assessments indicated that the condyle, ramus, and inferior border of the mandible were within normal limits. Similarly, the maxilla, hard palate, nasal cavity, and maxillary sinuses exhibited normal anatomical variations (Figure 4A). However, the mandibular alveolar bone appeared thin in regions with missing teeth. Periapical lesions were identified in the lower permanent incisors (Figure 4B).

Treatment plan

In this complex case, a multidisciplinary approach is essential to address the patient's unique needs. In the pedodontics phase, an individualized preventive plan has been implemented, which includes oral hygiene instructions, diet counseling, dental prophylaxis, topical fluoride application, and fissure sealants for deep retentive fissures. For the restorative phase, composite restorations were performed to address occlusal caries. Regular recall appointments every three months will be scheduled, and a referral to oral medicine will be made as necessary.

In the periodontics and endodontics aspects of care, consultations have been sought for the lower anterior teeth. Extraction of tooth #41 is recommended due to severe mobility and the risk of aspiration, with regular follow-up planned for the remaining teeth. Regarding prosthodontics, consultations have been sought for the management of multiple missing lower teeth. The decision has been made against a removable appliance due to severe bone loss and the potential for trauma to the gingiva and mucosa, considering the patient's lack of sensation and pain reflex.

Outcome

The outcome of the treatment was highly positive, as the patient started following up every week. The traumatic ulcers in the patient's oral cavity have completely healed, and their oral hygiene is now excellent. Furthermore, the patient has experienced significantly improved tooth function, speech, and enhanced self-esteem. It's important to note that while the patient may face the possibility of further tooth loss in the future, regular dental visits are expected to help delay this progression. The patient's guardian has a clear understanding of this and is committed to maintaining the patient's oral health through regular dental check-ups.

## Discussion

The presented case offers a compelling glimpse into the intricate clinical landscape of CIPA, shedding light on the multifaceted challenges it presents for both systemic health and oral well-being. This case underscores the importance of an interdisciplinary approach to understanding, diagnosing, and managing this complex condition.

Clinical implications of CIPA

CIPA, characterized by the absence of pain perception, anhidrosis, and intellectual disability, presents a unique set of clinical challenges [13]. The patient's profile aligns well with the classic presentation of CIPA, demonstrating self-inflicted injuries, developmental delays, and a history of recurrent fever episodes. Finger and nail biting is a common manifestation [14]. The oral findings of CIPA patients usually include an increased incidence of traumatic injuries. The constant feature of this disorder is self-mutilation, frequently involving the lips, buccal mucosa, teeth, and tongue [15]. In severe cases of CIPA, recurrent bone fractures and systemic infections were reported, resulting from self-mutilation and anhidrosis [3,4]. The lack of pain perception in CIPA can lead to inadvertent injuries and burns that often go unnoticed. Consequently, these injuries can become complicated by infections, including cellulitis, due to the absence of typical protective behavioral responses seen in individuals with normal pain perception [16].

Oral manifestations and dental challenges

The case further elucidates the intricate interactions between genetic factors and dental health within the context of CIPA. The presence of congenitally missing teeth and early exfoliation of primary teeth exemplifies the dental manifestations of CIPA [17]. The extraction of unrestorable teeth due to caries and mobility emphasizes the challenges of maintaining oral health without the guidance of pain sensations. Prevention of dental disease is very important in these patients, which is exacerbated by the limited ability to perceive and respond to dental pain, leading to consequences such as infection and tooth loss. Furthermore, irregular oral hygiene practices and the need for dental rehabilitation under general anesthesia highlight the comprehensive nature of dental care for individuals with CIPA [18]. The case underscores the importance of tailored dental interventions that incorporate both preventive education and specialized professional care. Several methods can be implemented for the prevention of oral mucosal injuries in CIPA patients, such as the elimination of sharp surfaces of the teeth (enameloplasty) or the addition of composite restorations [4]. The patient may require the use of mouth guards, tongue guards, or other appliances to prevent injury to the tongue. In severe cases, extraction of the offending teeth might be needed [19].

Dental mobility and implications

The intriguing finding of Grade II dental mobility in the permanent mandibular incisors is particularly noteworthy. While a cold test to assess dental pulp vitality was not feasible due to the patient's medical condition, the presence of altered dental mobility in a pain-insensitive environment accentuates the complexities of dental health in CIPA patients. The neuropathic nature of CIPA may contribute to dental movement in ways that differ from individuals with normal pain perception, further underscoring the need for innovative approaches to dental assessment and care in this population [13].

Pain management and anesthesia considerations

The intricate clinical dynamics of CIPA warrant thoughtful consideration of pain management strategies in routine dental procedures. While the absence of pain perception poses unique challenges, a balance must be struck in determining the role of local anesthesia. A notable contention arises regarding the necessity of local anesthesia in routine dental interventions, such as pulp treatments, given the lack of responsiveness to pain stimuli exhibited by individuals with CIPA [3,4]. Undoubtedly, the primary purpose of local anesthesia in dental procedures is pain alleviation, a fundamental aspect that CIPA patients inherently lack. This raises questions about the extent to which conventional local anesthesia is warranted for pain management in individuals who do not experience pain. However, it is imperative to recognize that local anesthesia holds additional significance beyond pain mitigation. Local anesthetics with vasoconstrictors are crucial for minimizing intraoperative bleeding and optimizing visualization, particularly during procedures like extractions [20]. Navigating the anesthetic needs of individuals with CIPA is further compounded when considering sedative or general anesthetic management [21]. While these approaches may be indicated in certain cases, they present increased challenges due to the heightened risk of complications such as regurgitation, aspiration, hyperthermia, and bradycardia [22]. This underscores the necessity for a meticulous and tailored approach to selecting anesthesia options, weighing the potential benefits against the inherent risks.

Interdisciplinary collaboration and future considerations

The challenges posed by this rare disorder necessitate an interdisciplinary approach that addresses genetic underpinnings, systemic implications, and specific dental considerations [23]. Collaboration among clinicians, geneticists, pediatricians, and dentists is paramount to holistically address the challenges posed by CIPA [13]. The development of specialized pain assessment tools for CIPA patients has the potential to revolutionize dental and medical care, enabling timely interventions and enhanced quality of life. Continued research and the sharing of insights among healthcare disciplines will be vital in advancing understanding and care for individuals with CIPA and related genetic disorders.

## Conclusions

The case of an 11-year-old girl with CIPA highlights the intricate challenges of this rare genetic disorder, revealing its impact on systemic health and oral well-being. The absence of pain perception, recurrent fever episodes, and self-inflicted injuries underscore its complex nature. The dental history emphasizes tailored interventions, addressing early exfoliation, dental infections, and the need for specialized rehabilitation. A cheerful demeanor amid developmental delay and the presence of ulcers reveals the paradox of CIPA's manifestations. Early diagnosis and specific dental care can help prevent self-inflicted orofacial trauma, emphasizing the role of proactive interventions to counter pain insensitivity. This case underscores the necessity of an interdisciplinary approach, innovative pain management, and comprehensive care to enhance the lives of individuals with CIPA.

## References

[1] Rosemberg S, Marie SKN, Kliemann S (1994). Congenital insensitivity to pain with anhidrosis (hereditary sensory and autonomic neuropathy type IV). Pediatric Neurology.

[2] Indo Y (2001). Molecular basis of congenital insensitivity to pain with anhidrosis (CIPA): mutations and polymorphisms in TRKA (NTRK1) gene encoding the receptor tyrosine kinase for nerve growth factor. Hum Mutat.

[3] Butler J, Fleming P, Webb D (2006). Congenital insensitivity to pain-review and report of a case with dental implications. Oral Surg Oral Med Oral Pathol Oral Radiol Endod.

[4] Bae C, Lee D, Kim J, Yang Y (2019). Dental management in a patient with congenital insensitivity to pain with anhidrosis: a case report. Journal of the Korean Academy of Pediatric Dentistry.

[5] Daneshjou K, Jafarieh H, Raaeskarami SR (2012). Congenital insensitivity to pain and anhydrosis (CIPA) syndrome; a report of 4 cases. Iran J Pediatr.

[6] Khaled B, Alzahayqa M, Jaffal A (2023). Identification of founder and novel mutations that cause congenital insensitivity to pain (CIP) in palestinian patients. BMC Med Genomics.

[7] Axelrod FB, Gold-von Simson G (2007). Hereditary sensory and autonomic neuropathies: types II, III, and IV. Orphanet J Rare Dis.

[8] Cascella M, Muzio MR, Monaco F, Nocerino D, Ottaiano A, Perri F, Innamorato MA (2022). Pathophysiology of nociception and rare genetic disorders with increased pain threshold or pain insensitivity. Pathophysiology.

[9] Indo Y (2012). Nerve growth factor and the physiology of pain: lessons from congenital insensitivity to pain with anhidrosis. Clin Genet.

[10] Axelrod FB, Chelimsky GG, Weese-Mayer DE (2006). Pediatric autonomic disorders. Pediatrics.

[11] Lee ST, Lee J, Lee M, Kim JW, Ki CS (2009). Clinical and genetic analysis of Korean patients with congenital insensitivity to pain with anhidrosis. Muscle Nerve.

[12] Pérez-López LM, Cabrera-González M, Gutiérrez-de la Iglesia D, Ricart S, Knörr-Giménez G (2015). Update review and clinical presentation in congenital insensitivity to pain and anhidrosis. Case Rep Pediatr.

[13] Indo Y (2020). NTRK1 Congenital Insensitivity to Pain With Anhidrosis. https://europepmc.org/article/NBK/nbk1769.

[14] Gao L, Guo H, Ye N (2013). Oral and craniofacial manifestations and two novel missense mutations of the NTRK1 gene identified in the patient with congenital insensitivity to pain with anhidrosis. PLoS One.

[15] Bodner L, Woldenberg Y, Pinsk V, Levy J (2002). Orofacial manifestations of congenital insensitivity to pain with anhidrosis: a report of 24 cases. Journal of dentistry for children.

[16] Wilkinson IB, Raine T, Wiles K, Goodhart A, Hall C, O'Neill H (2017). Oxford Handbook of Clinical Medicine. https://books.google.ae/books?hl=en&lr=&id=DZo6DwAAQBAJ&oi=fnd&pg=PP1&dq=Oxford+handbook+of+clinical+medicine&ots=wWfV5JEe9v&sig=52MO36p0-fgKunm7Ryv9blKeVC0&redir_esc=y#v=onepage&q=Oxford%20handbook%20of%20clinical%20medicine&f=false.

[17] Mostafa MI, Abdelkader MA, Abdelrahman MA (2021). Frequent genetic disorders associated with missing teeth and revisiting classification of anodontia: a retrospective study. Middle East Journal of Medical Genetics.

[18] Esmaeilzadeh N, Ashrafi MR, Shojaaldini Ardakani H, Seraj B, Aref P (2022). Hereditary autonomic neuropathy of the oral cavity and its management. Iran J Child Neurol.

[19] Kumar VA, Jaishankar H, Naik P (2014). Congenital insensitivity to pain: review with dental implications. Indian Journal of Pain.

[20] Kouvelas N, Terzoglou C (1989). Congenital insensitivity to pain with anhidrosis: case report. Pediatric Dentistry.

[21] Zlotnik A, Natanel D, Kutz R (2015). Anesthetic management of patients with congenital insensitivity to pain with anhidrosis: a retrospective analysis of 358 procedures performed under general anesthesia. Anesth Analg.

[22] Zlotnik A, Gruenbaum SE, Rozet I, Zhumadilov A, Shapira Y (2010). Risk of aspiration during anesthesia in patients with congenital insensitivity to pain with anhidrosis: case reports and review of the literature. J Anesth.

[23] Al Amroh HH, Reyes AL, Barret Austin Hillary J, Al Khaffaf WH (2020). Painless: a case of congenital insensitivity to pain in a 5-year-old male. Oxf Med Case Reports.

